# The effect of orally consumed *Lactuca sativa* syrup on human milk volume and weight gain in the preterm infant: a randomized controlled clinical trial

**DOI:** 10.1038/s41598-023-46441-0

**Published:** 2023-11-02

**Authors:** Niloufar Izaddoost, Leila Amiri-Farahani, Shima Haghani, Arash Bordbar, Asie Shojaii, Sally Pezaro

**Affiliations:** 1 Department of Reproductive Health and Midwifery, School of Nursing and Midwifery, Iran University of Medical Sciences, Tehran, Iran; 2 Department of Reproductive Health and Midwifery, Nursing and Midwifery Care Research Center, School of Nursing and Midwifery, Iran University of Medical Sciences, Tehran, 1996713883 Iran; 3 Department of Biostatistics, Nursing and Midwifery Care Research Center, Iran University of Medical Sciences, Tehran, Iran; 4 Shahid Akbarabadi Clinical Research Development Unit (ShACRDU), Iran University of Medical Sciences (IUMS), Tehran, Iran; 5 Research Institute for Islamic and Complementary Medicine, School of Persian Medicine, Iran University of Medical Sciences, Tehran, Iran; 6 The Research Centre for Healthcare and Communities, Coventry University, Coventry, UK; 7 The University of Notre Dame, Notre Dame, Australia

**Keywords:** Health care, Medical research

## Abstract

Human milk feeding can support premature infants to thrive. Yet those with premature infants can be challenged in human milk production. Considering this, and the use of potentially harmful human milk enhancers, the present study was conducted with the aim of determining the effect of orally consumed *Lactuca sativa* (*L. sativa*) syrup (lettuce extract) on human milk volume and subsequent weight gain in the preterm infant. Extracts from lettuce and other plants such as silymarin are already evidenced to be safe for use during lactation and have other therapeutic effects in humans. Yet this is the first study of its kind. This parallel randomized clinical trial included lactating participants with their preterm infants who were born at < 32 weeks' gestation and admitted to an intensive care unit. Convenience sampling was used to recruit participants. Eligible participants were allocated to groups randomly: intervention (n = 47), placebo (n = 46), and control (n = 47). The intervention group received one tablespoon of *Lactuca sativa* (*L. sativa*) syrup, and the placebo group received one tablespoon of placebo syrup 3 times a day for 1 week. Those in the control group did not receive any herbal or chemical milk-enhancing compounds. Routine care was provided to all three groups. Participants recorded their milk volume for 7 days in a daily information recorder form. Infant weight was measured prior to the intervention, and on the third, fifth and seventh days of the intervention period. There was a statistically significant difference observed in the adjusted mean volume of milk on the fourth and fifth days between the intervention, placebo, and control groups (P < 0.05). The adjusted mean milk volume of those in the intervention group on the first day was significantly higher than those in the control group and those in the placebo group. On the second day, the adjusted mean milk volume of those in the intervention group was higher than in those from the control group; and on the fourth day it was higher than in those from both the control and placebo groups; on the fifth day it was higher than in those in the placebo group; on the sixth day it was higher than in those in the control group and on the seventh day it was higher than in those in the control group (P < 0.05). There was no statistically significant difference in terms of the mean changes (with or without adjustment) in the weight of preterm infants between any of the groups. *Lactuca sativa* (*L. sativa*) syrup increases the volume of human milk production and no specific side effects have been reported in its use. Therefore, *Lactuca sativa* syrup can be recommended for use as one of the compounds that increase human milk volume.

## Introduction

Human milk feeding is considered beneficial to both feeders and their infants^1,2^ .Yet in 2018, only 42% of infants worldwide began human milk feeding during the first hour of birth^3,4^ .A key global infant feeding goal is that at least 50% of children fewer than 6 months old be exclusively fed human milk by 2025^5^ .Close to this in Iran, the results of a systematic review and meta-analysis reported an overall prevalence of exclusive human milk feeding at 49%^6^.

The positive effects of human milk feeding, especially upon premature infants^7^ and the reduction of diseases and complications of prematurity are well known^8,9^. Human milk increases the IQ of premature infants^10^. It reduces the risk of necrotizing enterocolitis, retinopathy of prematurity^11^, along with late-onset sepsis, chronic lung disease, and pre hospitalization after discharge from the neonatal intensive care unit^11,12^. However, those birthing preterm infants may not produce enough human milk to provide these benefits^9^. Thus, it will be important to explore ways in which to increase milk production in these populations.

Premature birth in particular is a risk factor in delayed or insufficient human milk feeding where high doses of human milk provide the greatest protection^13^. Premature infants are also significantly more likely to be resuscitated at birth and require respiratory and nutritional support causing parental separation and delayed onset of human milk feeding^14^. Parental separation from the premature infant has a negative effect on the continuous production of milk^15^. Additionally, premature infants have an immature physiological system and neurological development^16^, are exposed to low human milk consumption due to poor sucking and sleepiness in addition to delayed initiation of human milk feeding^17^ .Many are unable to provide human milk to their infants due to illness and stress caused by the clinical condition of their infant^13,18–20^. To overcome such challenges, new interventions are required.

Those who do not produce enough human milk and do not respond to counseling can use chemical and herbal milk additives to stimulate human milk production^21^chemical drugs that are dopamine antagonists such as domperidone and metoclopramide are associated with many side effects^18,22–24^. To increase human milk production, many also turn to herbal supplements despite limited scientific evidence on their efficacy and safety^25^ .

Using a combination of milk-enhancing plants such as fennel, black seed and cumin can be effective in improving human milk production^26^. Moreover, Ozalkaya et al. provided evidence that the consumption of milk-enhancing herbal tea containing 1% nettle and six other plants increases human lactation. Yet this was not found to have a significant effect on the weight gain of premature infants and the serum levels of prolactin^27^. Whilst Fenugreek has been found to increase milk production after childbirth and increase the weight of infants in the first week of life^28^, it can cause hypoglycemia and gastrointestinal discomfort. Concerns related to licorice and fennels include lethargy, hypotonia and vomiting in infants if a large amount of herbal compounds is consumed^29^. Thus, it will also be important to ensure that any interventions promoted to increase human milk production have minimal side effects.

Lettuce has a high nutritional value, and its medicinal properties have been widely reported throughout the world^30^. For example, due to the presence of lignan (one of the main components of the phytoestrogen family) and its precursors, lettuce has already been found to have a protective effect against breast cancer in premenopausal cisgender women and against esophageal cancer in humans^31,32^. Several other food compositions which include lettuce have also been found to increase human milk production^33–36^, though it is yet to be tested exclusively. Lettuce specifically contains phytoestrogens^34^, flavonoid compounds^37^ and lignans. Considering the above, we hypothesized that lettuce may have milk-enhancing properties, particularly due to the presence of the phytoestrogens within it.

Lettuce is also known to interact with opioid receptors^38,39^ and opioids, by acting on mast cells and causing their degranulation, histamine release, and vasodilation^40^. Furthermore, as lettuce has diuretic, laxative, moisturizing and thirst-quenching effects^40–42^. It is also known broadly to be one of the things that can increase milk production^33,35,36,43,44^. Nevertheless, its effect upon human milk production is yet to be tested exclusively.

Considering this, alongside its known therapeutic effects, availability and affordability its use was considered acceptable and safe in this context. Yet no clinical trial has yet to explore the milk-enhancing effects of lettuce syrup in humans, particularly in human milk feeding^45^. In light of the above, the present study aimed to determine the effect of orally consumed *Lactuca sativa* (*L. sativa*) syrup (lettuce extract) on human milk volume and subsequent weight gain in the preterm infant.

## Methods

### Participants and trial design

The current study was conducted as a parallel randomized clinical trial with intervention, placebo, and control groups**.** Reporting of this study has been done in accordance with the Consolidation Standards of Reporting Trials (CONSORT) statement^46^. It was funded by Iran University of Medical Sciences in October 2021. The study’s protocol was registered on 14–12-2021 in the clinical trial center, code IRCT20180427039436N12. Study participants were lactating with preterm infants who were born at < 32 weeks' gestation and admitted to the neonatal intensive care unit of Shahid Akbarabadi Hospital affiliated to Iran University of Medical Sciences in Tehran, Iran. Notably, some of these were admitted from surrounding hospitals.

#### Participant inclusion criteria

Participants were required to be at least 18 years old, able to read and write, willing to exclusively feed their infants with human milk, willing to pump their human milk through an electronic pump from the third day of the postpartum period and attend the hospital during the study period.

#### Infant inclusion criteria

Preterm infants with a birth age of < 32 weeks gestation (according to ultrasound), hospitalized in the neonatal intensive care unit and fed through an oral-gastric or nasogastric tube were included.

#### Participant exclusion criteria

Participants smoking, using alcohol, narcotics, neuropsychiatric drugs and/or any drug or herbal combination used to stimulate human milk production (galactagogues), those with infertility, infectious diseases transmitted through human milk feeding (e.g. HIV and active pulmonary tuberculosis), and those with previous breast surgery and/or problems such as abscess, inverted nipple, mastitis, or cancer were excluded from participation.

#### Infant exclusion criteria

Preterm infants resulting from twins or multiple births, and/or with congenital abnormalities (e.g., cleft lip/palate) and/or prescribed supplements were excluded from participation.

#### Withdrawal criteria

Participants could withdraw from the study at any time without declaring a reason. Participants were withdrawn from the study if they were consuming < 10% of the *Lactuca sativa* (*L. sativa*) syrup required; infants not consuming human milk exclusively (e.g.., using Human milk banks or artificial milk substitutes); using a pump < 10% of the time/amount recommended; using any drug or other herbal combination to stimulate milk production; experiencing allergic reactions; unable to commence human milk feeding.

#### Data collection/recruitment

Sampling was done continuously over a period of 7 months from January to August 2021. After describing the study and its objectives, written informed consent was obtained from participants who met the study entry criteria and agreed to participate.

Confirmed participants were allocated to three groups (1) intervention; (2) placebo and (3) control using block randomization via the website https://www.sealedenvelope.com/ and were followed up for 7 days. The size of each block was twice the number of groups (six groups in each block). The information required to achieve random allocation and concealment of research samples included the number of treatment groups, the number of blocks, the size of each block, and the number of individuals participating in the study. Input of this information resulted in a two-digit randomization code and a number for each sample being generated. This code clearly indicated which block the sample was from and which group it belonged to. An epidemiologist external to the research team prepared the randomization list and kept it concealed to hide the allocation from the team. Blinding was not possible due to the inclusion of a control group.

The volume of human milk was recorded as one of the main results of the study by the participants in the daily information recorder form. The weight of the infants was also measured and recorded by one of the neonatal intensive care unit staff, who was responsible for weighing the infants. Statistical analysis was performed by a statistician who was not aware of the content presented for the studied groups and their allocation.

### Description of intervention and comparator groups

Participants in the intervention group received 240 ml of *Lactuca sativa* syrup orally (Standardization of this syrup: standardized based on total phenol, containing at least 10 mg per 10 g of syrup). Participants in the placebo group received a placebo syrup orally, and prepared based on the base formula of the syrup (the extract used for the placebo of *Lactuca sativa* syrup was sugar). Participants in both intervention and placebo groups were advised to take one tablespoon three times a day, half an hour after breakfast, lunch, and dinner for 1 week. The researcher's phone number was given to participants in case of allergic reactions or complications during the study. Participants in the control group did not receive any herbal or chemical formulas to increase lactation. The intervention period was limited to 7 days as typically after this time, the preterm infant is fed directly from a human (rather than pumped human milk), which makes it impossible to accurately record human milk volume.

In order to simulate the appearance of placebo with *Lactuca sativa* syrup, both types of syrup were prepared and packaged by "Senabel Drug" pharmaceutical company. In order to avoid bias and increase the validity of results, participants, the researcher, and the statistician remained unaware of the content of the syrups given to both the intervention and placebo groups.

Participants in all groups used an electronic shower milk pump (Medela) at least 6 times a day (3–4 h after waking up from 8 a.m.) during the 7-day period to extract their human milk. Excess milk was stored in the milk bank of Shahid Akbarabadi Hospital.

### Assessment of trial variables

The variables of this study were measured as follows:

#### Individual characteristics

Individual characteristics were collected in relation to both parents and the infant. Maternal characteristics included age, body mass index, level of education, occupation, whether the pregnancy was planned, number of previous births, history of abortion, mode of birth, type of anesthesia or anesthesia in case of caesarean section, chronic diseases, use of drugs during pregnancy, use of steroids, the number of possible doses of steroids and the use of iron and complementary drugs used. Paternal characteristics included level of education, occupation, and socio-economic status. The characteristics of the infant included weight, height, head circumference, gestational age at birth based on ultrasound, age upon entry to the study, infant sex assigned at birth, cause of infant hospitalization, and mode of infant feeding. Maternal and paternal information was self-recorded by adult participants. Infant participant data was extracted from medical records.

#### Daily information recorder form as primary outcome

A daily information form was used for the daily recording of the time spent on the pump, frequency of using the pump, time spent skin-to-skin with the infant (kangaroo care), frequency of liquids consumed based on the number of glasses per day, consumption of foods with milk-enhancing properties such as lettuce, basil leaves, dill, fenugreek, carrot juice, spinach, sesame, fennel, the type and amount of any serums injected into the infant and volume of pumped milk at the end of each day. All adult participants entered the information manually and daily during the period of 7 days into the daily information recorder form. The daily data in relation to serum therapy was recorded by the researcher.

#### Infant weight recorder form as a primary outcome

Infant weight was measured on the first day (before the intervention), the third, the fifth and the seventh day shortly following the intervention period using a Seka digital scale made in Germany. Measurements were performed and recorded prior to feeding and after bowel/urinary outputs during the morning shift.

#### Medication side effects recorder form

This form was completed by the researcher in partnership with participants in order to investigate the side effects of the syrup, including hives, itching, heartache, heartburn, and drowsiness during the period of using the syrup.

### Sample size

To determine the minimum required sample size at the confidence level of 95% and the power of the test is 80% and the accuracy is 9, after quantification in the following formula, the minimum sample size was calculated to be 39 participants in each group. This was then estimated to be 48 participants in each group after taking into account a 20% sample drop out rate^47^ .Ultimately, the total number of participants in this study was estimated to be 144.

Sample size for our other research variable (infant weight), was calculated from Turkyılmaz et al.'s article that calculated weight changes in which 15 infants were allocated to each group^48^. Therefore, the variable of human milk volume was used for calculation to determine the sample size overall.

$$ n = \frac{{\left( {z_{{1 - \alpha /2}}  + z_{{1 - \beta }} } \right)^{2}  \times \left( {s_{1}^{2}  + s_{{2}}^{2} } \right)}}{{d^{2} }} $$

$${z}_{0.975}=1.96 \;\; {z}_{0.8}=0.84$$

$${s}_{1}=18.8 \;\; {s}_{2}=7$$

$$d=9$$

$$n=\frac{({1.96+0.84)}^{2}\times ({18.8}^{2}+{7}^{2})}{{9}^{2}}\approx 39$$

### Ethics

The protocol of the present study was approved by the Ethics Committee of Iran University of Medical Sciences with the ethical code IR.IUMS.REC.1400.671. All participants were fully informed about the goals and process of the study and written consent was obtained from them. The information obtained during the study process remained strictly confidential. All participants were informed that they could withdraw from the study at any time without declaring a reason. All services were completely free. All data were anonymously entered into the data systems hosted by the lead institution. All participants were given the researcher's phone number at the beginning of the study to ensure that support was available at all times for any reason (e.g., side effects).

### Statistical analysis

Statistical analysis of data and comparison of groups was done using one-way analysis of variance (ANOVA) for quantitative variables such as maternal age and body mass index (BMI), number of previous births, number of steroid injections, infant birth weight, height, head circumference, gestational age at birth based on ultrasound and age upon entry to the study, frequency of liquids consumed based on the number of glasses per day, frequency of pump usage, time spent on the pump, time spent skin-to-skin with the infant, number of infant serum injections, amount of milk pumped at the end of each day and the weight of infants on the first (before intervention), third, fifth and seventh days after participating in the study. Fisher's exact test was used for variables relating to paternal occupation and the reason for the infant's admission to the hospital. The Chi-Square test was used for qualitative variables such as educational levels, maternal occupation, whether the pregnancy was planned, mode of birth, abortion history, chronic diseases, drug use during pregnancy, maternal use of iron and complementary drugs, the socio-economic status of the family and the infant’s sex assigned at birth, the consumption of foods with milk-increasing properties and type of infant serum injections.

The significance of variables such as the mode of birth, the infant's birth weight, the infant's height, intrauterine growth restriction, the type and number of infant serum injections was calculated. Yet in order to adjust these variables from covariance analysis for frequency of pump usage, time spent on pump, time spent skin-to-skin with the infant, the number of infant serum injections, the amount of milk pumped at the end of each day and the weight of infants on the first (before the intervention), third, fifth and seventh day shortly after the study intervention period, the logistic regression model was used.

Analysis of variance with repeated measures was used to compare the amount of human milk pumped at the end of each day and the weight of infants on the first (before the intervention), third, fifth and seventh days shortly after the intervention period of the study. The data were analyzed using SPSS software version 23 (IBM Corp, US). The significance level of the tests was considered to be < 0.05. The eta square index was considered to measure the effect size.

## Results

**Figure 1:**
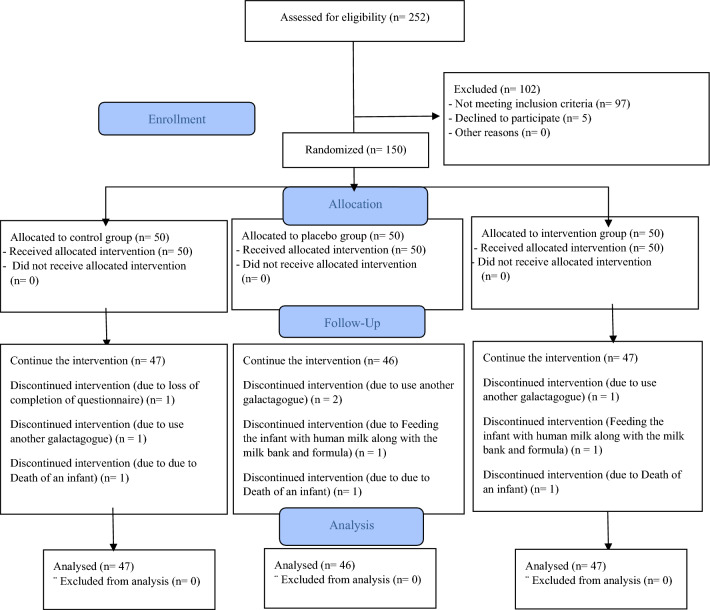
Enrolment of participants into three groups of intervention, placebo, and control.

Overall, 150 lactating adults with preterm infants participated in the study (50 in the intervention group, 50 in the placebo group and 50 in the control group). One lactating participant and infant were withdrawn from the study due to lack of questionnaire completion, 4 were withdrawn as they chose to use additional galactagogues, 2 were withdrawn as they chose to feed their infants with additional human milk from a bank and formula milk, and 3 were withdrawn due to due to the death of an infant. Overall, (n = 140) (47 in the intervention group, 46 in the placebo group and 47 in the control group) remained in the study and generated data to be analyzed statistically (Fig. 1). No adverse side effects were reported by the participants.

The three groups did not demonstrate a statistically significant difference in terms of demographics, maternal, paternal, and infant baseline data. There was a statistically significant difference only in mode of birth, infant's weight, infant's height, and intrauterine growth restriction among the causes of hospitalization in the three groups (P < 0.05) (Table 1). Only spinal anesthesia was used during childbirth, and all participants had received injectable steroids. In all preterm infant participants, oral-gastric tube feeding, and respiratory distress syndrome were identified reasons for their hospitalization.

**Table 1:** Demographics and baseline characteristics of study participants and comparisons between intervention, placebo, and control groups.

Variables		Intervention group (n = 47)	Placebo group (n = 46)	Control group (n = 47)	P-value
Maternal age (year), (mean ± SD)		30.17 ± 5.94	30.43 ± 6.78	29.66 ± 7.49	0.854
Maternal BMI (kg/m^2^), (mean ± SD)		25.17 ± 4.77	25.14 ± 3.40	26.31 ± 3.26	0.257
Maternal education, n (%)					
High school		15 (31.9)	21 (45.65)	24 (51.1)	0.410
Diploma		26 (55.3)	21 (45.65)	18 (38.3)	
University		6 (12.8)	4 (8.7)	5 (10.6)	
Maternal occupation, n (%)					
Housewife		41 (87.2)	42 (91.3)	42 (89.4)	0.817
Employed		6 (12.8)	4 (8.7)	5 (10.6)	
Pregnancy planned, n (%)					
Yes		33 (83)	39 (84.8)	38 (83)	0.166
No		14 (17)	7 (15.2)	8 (17)	
Number of previous births (mean ± SD)					
1		22 (46.8)	19 (41.3)	21 (44.7)	0.477
2		20 (42.6)	17 (37.0)	17 (36.2)	
≥ 3		5 (10.6)	10 (21.7)	9 (19.1)	
Mode of birth, n (%)					
Vaginal birth		11 (23.4)	13 (28.3)	29 (61.7)	** < 0.001**
Birth via caesarean section		36 (76.6)	33 (71.7)	18 (38.3)	
History of abortion, n (%)					
Yes		16 (34.0)	12 (26.1)	10 (21.3)	0.372
No		31 (66.0)	34 (73.9)	37 (78.7)	
Maternal chronic diseases, n (%)					
Yes		15 (31.9)	15 (32.6)	7 (14.9)	0.089
No		32 (68.1)	31 (67.4)	40 (85.1)	
Maternal drug use during pregnancy, n (%)					
Yes		15 (31.9)	15 (32.6)	7 (14.9)	0.089
No		32 (68.1)	31 (67.4)	40 (85.1)	
Maternal use of iron and complementary drugs, n (%)					
Yes		47 (100)	45 (97.8)	46 (97.9)	0.496
No		0 (0)	1 (2.2)	1 (2.1)	
Number of steroid injections, n (%)					
1		14 (29.8)	16 (34.75)	9 (19.1)	0.729
2		25 (53.2)	21 (45.65)	35 (74.5)	
≥ 3		8 (17.0)	9 (19.6)	3 (6.4)	
Paternal occupation, n (%)					
Employee		8 (17.06)	5 (10.9)	11 (23.45)	0.374
Manual worker		16 (34.04)	12 (26.1)	16 (34.00)	
Self- employed		23 (48.90)	27 (58.7)	19 (40.45)	
Unemployed		0 (0.00)	2 (4.3)	1 (2.10)	
Paternal education, n (%)					
High school		21 (44.7)	26 (56.5)	18 (38.3)	0.314
Diploma		20 (42.5)	14 (34.45)	18 (38.3)	
University		6 (12.8)	6 (13.0)	11 (23.4)	
Economic status of the family, n (%)					
Desirable		12 (25.5)	10 (21.7)	10 (21.26)	0.292
Relatively		28 (59.6)	28 (60.9)	35 (74.50)	
Undesirable		7 (14.9)	8 (17.4)	2 (4.24)	
Infant's weight, (gr) (mean ± SD)		1464.34 ± 328.29	1344.15 ± 307.37	1220.32 ± 154.51	** < 0.001**
Infant's height, (cm) (mean ± SD)		39.71 ± 2.97	39.73 ± 2.79	38.37 ± 2.05	**0.018**
Head circumference of infant, (cm) (mean ± SD)		29.18 ± 2.24	28.79 ± 2.08	29.01 ± 2.05	0.680
Gestational age at birth, (wk) (mean ± SD)		29.91 ± 1.26	29.72 ± 1.25	29.55 ± 1.03	0.341
Neonatal age upon entry to the study, (wk) (mean ± SD)		7.32 ± 4.09	8.15 ± 4.56	8.09 ± 3.38	0.543
Infant sex assigned at birth, n (%)					
Male		29 (61.7)	21 (45.7)	22 (46.8)	0.223
Female		18 (38.3)	25 (54.3)	25 (53.2)	
Cause of infant hospitalization, n (%)					
IUGR	Yes	3 (6.4)	7 (15.2)	14 (29.8)	**0.010**
	No	44 (93.6)	39 (84.8)	33 (70.2)	
PDA	Yes	2 (4.3)	1 (2.2)	5 (10.6)	0.275
	No	45 (95.7)	45 (97.8)	42 (89.4)	
IVH < 3	Yes	2 (4.3)	1 (2.2)	3 (6.4)	0.871
	No	45 (95.7)	45 (97.8)	44 (93.6)	
Jaundice	Yes	4 (8.5)	2 (4.3)	2 (4.3)	0.731
	No	43 (91.5)	44 (95.7)	45 (95.7)	
Cyanosis	Yes	0 (0.0)	0 (0.0)	2 (4.3)	0.329
	No	47 (100.0)	46 (100.0)	45 (95.7)	

SD: standard deviation, BMI: Body Mass Index, IUGR: intra uterine growth restriction, PDA: patent ductus arteriosus, IVH: intra ventricular hemorrhage, D.W: dextrose water.

Significant values are in bold.

The three groups did not demonstrate a statistically significant difference in terms of maternal, paternal, and infant characteristics. A statistically significant difference was seen in the type of serum injected into the preterm infants and the mean frequency of using the pump on the seventh day (P < 0.05) (Table 2).

**Table 2:** Recorded daily information and infant weight in three groups of study.

Variables			Intervention group (n = 47)	Placebo group (n = 46)	Control group (n = 47)	P-value
Frequency of tea and liquids based on the number of glasses consumed during the day (mean ± SD)	Day 1		5.43 ± 2.08	5.54 ± 1.72	5.21 ± 1.64	0.676
	Day 2		5.57 ± 1.92	5.59 ± 1.78	5.06 ± 1.84	0.299
	Day 3		5.83 ± 2.28	5.61 ± 2.02	5.19 ± 1.63	0.295
	Day 4		5.62 ± 2.34	5.37 ± 1.85	4.81 ± 1.51	0.120
	Day 5		5.81 ± 2.08	5.63 ± 1.85	5.19 ± 1.62	0.259
	Day 6		5.96 ± 2.26	6.00 ± 1.96	5.21 ± 1.66	0.100
	Day 7		5.64 ± 2.05	5.70 ± 1.89	5.13 ± 1.55	0.265
Use of foods with milk-increasing properties, n (%)	Day 1	Yes	29 (61.7)	21 (45.7)	30 (63.8)	0.154
		No	18 (38.3)	25 (54.3)	17 (36.2)	
	Day 2	Yes	30 (63.8)	22 (47.8)	30 (63.8)	0.196
		No	17 (36.2)	24 (52.2)	17 (36.2)	
	Day 3	Yes	31 (66.0)	34 (73.9)	25 (53.2)	0.109
		No	16 (34.0)	12 (26.1)	22 (46.8)	
	Day 4	Yes	35 (74.5)	36 (78.3)	27 (57.4)	0.065
		No	12 (25.5)	10 (21.7)	20 (42.6)	
	Day 5	Yes	39 (80.9)	27 (58.7)	33 (70.2)	0.066
		No	9 (19.1)	19 (41.3)	14 (29.8)	
	Day 6	Yes	36 (76.6)	32 (69.6)	28 (59.6)	0.733
		No	11 (23.4)	14 (30.4)	12 (25.5)	
	Day 7	Yes	37 (78.7)	32 (69.6)	28 (59.6)	0.132
		No	10 (21.3)	14 (30.4)	19 (40.4)	
Frequency of using pump (mean ± SD)	Day 1		6.40 ± 0.61	6.33 ± 0.56	6.30 ± 0.59	0.344
	Day 2		6.53 ± 0.62	6.50 ± 0.58	6.40 ± 0.61	0.548
	Day 3		6.53 ± 0.65	6.63 ± 0.71	6.55 ± 0.68	0.601
	Day 4		6.72 ± 0.74	6.57 ± 0.62	6.49 ± 0.62	0.235
	Day 5		6.60 ± 0.53	6.41 ± 0.54	6.34 ± 0.60	0.198
	Day 6		6.64 ± 0.76	6.61 ± 0.64	6.36 ± 0.56	0.101
	Day 7		6.55 ± 0.71	6.41 ± 0.54	6.30 ± 0.50	**0.046**
Time spent on pump (min) (mean ± SD)	Day 1		149.85 ± 31.67	156.11 ± 37.56	138.49 ± 24.29	0.092
	Day 2		155.53 ± 30.73	156.83 ± 30.64	137.15 ± 20.62	0.051
	Day 3		149.94 ± 29.12	160.63 ± 34.39	138.04 ± 27.00	0.422
	Day 4		152.49 ± 35.42	151.93 ± 37.84	137.77 ± 26.35	0.393
	Day 5		144.72 ± 35.15	143.78 ± 33.03	127.15 ± 28.24	0.325
	Day 6		144.09 ± 33.27	140.96 ± 28.87	125.11 ± 28.57	0.527
	Day 7		143.98 ± 34.50	136.33 ± 26.85	119.38 ± 30.21	0.165
Time spent skin-to-skin with the infant (min) (mean ± SD)	Day 1		17.66 ± 7.21	16.20 ± 5.29	19.15 ± 6.62	0.056
	Day 2		17.45 ± 5.88	17.07 ± 7.27	19.26 ± 6.50	0.692
	Day 3		18.94 ± 7.06	18.59 ± 7.27	20.32 ± 7.02	0.867
	Day 4		19.47 ± 8.42	19.02 ± 8.79	20.21 ± 8.27	0.360
	Day 5		19.68 ± 6. 94	18.04 ± 10.46	20.21 ± 8.33	0.975
	Day 6		20.43 ± 7.43	19.24 ± 9.54	20.96 ± 7.27	0.775
	Day 7		22.02 ± 8.76	20.76 ± 9.77	20.66 ± 8.45	0.161
Type of serum injected into the infant, n (%)						
D.W 5%			9 (19.1)	21 (45.7)	27 (57.4)	** < 0.001**
D.W 10%			38 (80.9)	25 (54.3)	20 (42.6)	
Number of infant serum injections (cc) (mean ± SD)	Day 1		162.19 ± 50.08	141.37 ± 43.14	138.21 ± 35.76	0.903
	Day 2		162.49 ± 52.03	135.63 ± 43.15	135.23 ± 33.65	0.799
	Day 3		166.00 ± 50.73	132.30 ± 44.82	122.89 ± 31.31	0.403
	Day 4		162.60 ± 50.73	127.04 ± 48.13	115.00 ± 29.44	0.958
	Day 5		151.87 ± 47.77	122.70 ± 42.62	108.02 ± 26.66	0.244
	Day 6		147.40 ± 53.10	119.39 ± 47.92	99.11 ± 23.28	0.996
	Day 7		140.85 ± 49.49	111.13 ± 45.66	97.02 ± 23.81	0.449

Significant values are in bold.

Despite compliance with randomization, the following variables: mode of birth, infant weight and height, Intrauterine Growth Restriction (IUGR) and the type of serum injected into the infant were significantly different between the three groups. We controlled this effect by ensuring that the analysis was performed once with the presence of a moderation effect and once without.

**Figure 2:**
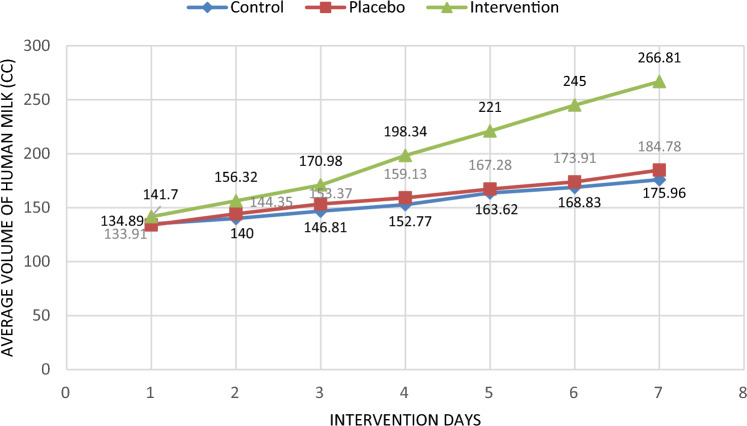
The mean daily volume of milk during 1 week of study, in the intervention, placebo and control groups.

**Figure 3:**
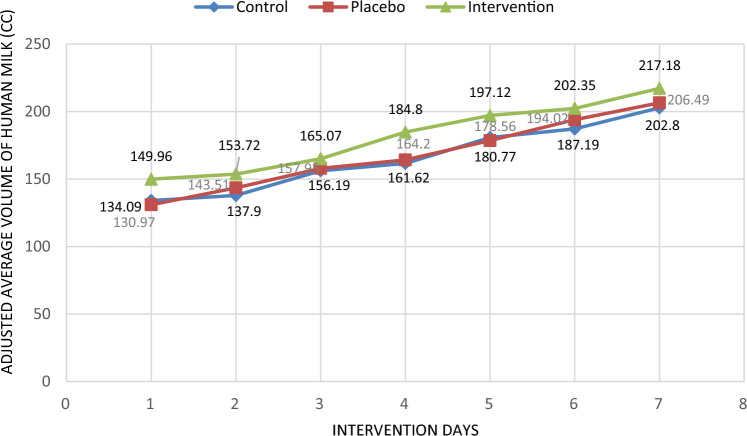
Adjusted mean daily milk volume during 1 week of study, in the intervention, placebo and control groups (adjusting the variables of mode of birth, birth weight of the infant, intrauterine growth delay and volume of pumped milk the previous day).

**Figure 4:**
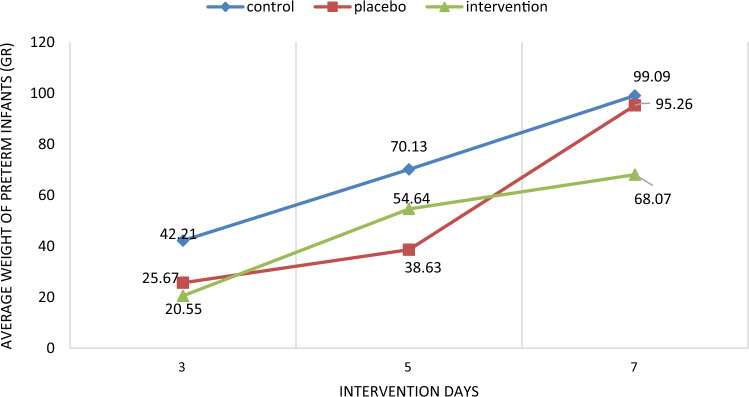
The mean weight changes of preterm infants in the three groups of intervention, placebo and control on the third, fifth and seventh days.

**Figure 5:**
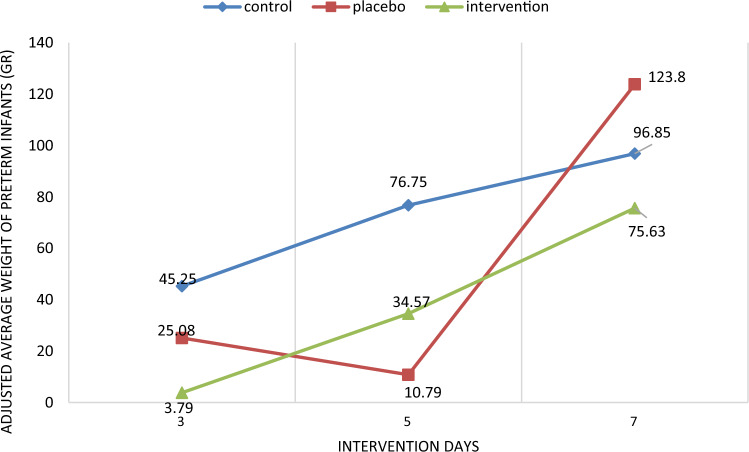
Adjusted mean weight changes of preterm infants in the three groups of intervention, placebo and control on the third, fifth and seventh days.

In the between group comparison there was a statistically significant difference observed (p < 0.05) between the three groups in relation to the mean volume of pumped milk on the second, third, fourth, fifth, sixth and seventh days. After adjusting the following variables: mode of birth, infant's weight, IUGR and the volume of milk pumped the previous day, there was a statistically significant difference (p < 0.05) observed in the adjusted mean volume of milk pumped on the fourth and fifth days. There was no statistically significant difference observed between the three groups in terms of preterm infant weight at the end of the intervention (7th day). Even after adjusting the variables of infant's weight and infant's height, IUGR and the type of serum injected into the infant, the weight of preterm infants at the end of the intervention (the seventh day) did not have a statistically significant difference (Table 3, Figs. 2, 3, 4, 5).

**Table 3:** Amount of pumped (± adjustment) milk at the end of each day and weighing of preterm infants (± adjustment).

Variables		Without adjustment				With adjustment			
		Intervention group (n = 47)	Placebo group (n = 46)	Control group (n = 37)	P-value^a,b^^,c,d^	Intervention group (n = 47)	Placebo group (n = 46)	Control group (n = 47)	P-value^a,b^^′,c′,d^^′^
Amount of pumped milk at the end of each day (cc) (mean ± SD)	Day 1	141.70 ± 24.89	133.91 ± 20.73	134.89 ± 14.72	0.140, 0.111, 0.105, 0.793	149.96 ± 6.45	130.97 ± 4.25	134.09 ± 3.41	0.050, **0.040**, **0.029,** 0.467
	Day 2	156.32 ± 33.36	144.35 ± 27.33	140.00 ± 15.70	**0.010, 0.003,** 0.062, 0.352	153.72 ± 6.04	143.51 ± 3.94	137.90 ± 3.15	0.071, **0.009,** 0.249, 0.313
	Day 3	170.98 ± 41.24	153.37 ± 31.83	146.81 ± 21.78	**0.001, 0.001, 0.024,** 0.248	165.07 ± 6.22	157.95 ± 4.06	156.19 ± 3.31	0.482, 0.333, 0.496, 0.621
	Day 4	198.34 ± 51.21	159.13 ± 26.29	152.77 ± 25.25	** < 0.001, < 0.001, < 0.001,** 0.237	184.80 ± 6.31	164.20 ± 4.07	161.62 ± 3.32	**0.008, 0.010, 0.016,** 0.541
	Day 5	218.09 ± 60.40	167.28 ± 33.11	163.62 ± 29.52	** < 0.001, < 0.001, < 0.001,** 0.574	197.12 ± 6.22	178.56 ± 3.96	180.77 ± 3.27	**0.048,** 0.072, **0.040,** 0.843
	Day 6	245.00 ± 65.83	173.91 ± 32.91	168.83 ± 36.13	** < 0.001, < 0.001, < 0.001,** 0.480	202.35 ± 5.51	194.02 ± 3.79	187.19 ± 3.12	0.077, **0.040,** 0.250, 0.152
	Day 7	266.81 ± 68.10	184.78 ± 36.10	175.96 ± 39.44	** < 0.001, < 0.001, < 0.001,** 0.264	217.18 ± 5.51	206.49 ± 3.46	202.80 ± 2.91	0.099, **0.040,** 0.152, 0.331
Weighing of preterm infants (gr) (mean ± SD)	Day 3	20.55 ± 50.99	25.67 ± 26.23	42.21 ± 18.85	**0.009, 0.008,** 0.543,** 0.001**	3.79 ± 11.17	25.08 ± 7.11	45.25 ± 6.30	**0.004, 0.008,** 0183,** 0.001**
	Day 5	54.64 ± 62.29	38.63 ± 101.43	70.13 ± 22.78	0.097, 0.115, 0.360, **0.041**	34.57 ± 20.94	10.79 ± 13.33	76.75 ± 11.81	**0.001,** 0.118, 0.458,** < 0.001**
	Day 7	68.07 ± 29.81	95.26 ± 145.30	99.09 ± 28.81	0.894, 0.403, 0.822, 0.860	75.63 ± 29.54	123.80 ± 18.81	96.85 ± 16.66	0.328**,** 0.422, 0.269**,** 0.349

^a,b^^,c,d^Repeated measurement test was used for comparison of the three groups and Tukey post hoc test was used for two- by-two comparison of groups.

^b^^′,c′,d′^Repeated measurement test was used for comparison of the three groups and Bonferroni post hoc test was used for two- by-two comparison of groups.

^a^Comparison of the three groups, ^b^comparison of Intervention vs. Control, ^c^comparison of Intervention vs. Placebo, ^d^comparison of Placebo vs. Control, ^b′^comparison of Intervention vs. Control, ^c′^comparison of Intervention vs. Placebo, ^d′^comparison of Placebo vs. Control.

Significant values are in bold.

Results from the analysis of variance with repeated measures for within-group comparisons demonstrated that in all groups the mean volume of pumped milk was statistically significant (p < 0.001) at least one time. Two-by-two comparisons in all times for three groups are presented in Table 4.

**Table 4:** Comparison of within-group of volume of pumped milk in three groups of study.

Variables	Comparison of the times^a^	Comparison between seventh and first day	Comparison between seventh and second day	Comparison between seventh and third day	Comparison between seventh and fourth day	Comparison between seventh and fifth day	Comparison between seventh and sixth day
Intervention group	< 0.001	< 0.001	< 0.001	< 0.001	< 0.001	0.002	< 0.001
Placebo group	< 0.001	< 0.001	< 0.001	< 0.001	< 0.001	< 0.001	< 0.001
Control group	< 0.001	< 0.001	< 0.001	< 0.001	< 0.001	0.002	0.001

^a^Repeated measurement test was used for comparison of the times and Bonferroni correction test was used for two-by-two comparison of times in each group separately. All of the numbers are P-value.

The results of analysis of variance with repeated measurements for within-group comparison showed that the mean weight of preterm infants on the seventh day in the intervention and control groups was significantly higher than the fifth day, the third day and the first day (before the intervention) (P < 0.001) and in the placebo group it was significantly higher than the third day (P = 0.01) and the first day (before the intervention) (P < 0.001). The mean weight of preterm infants on the fifth day in the intervention and control groups was significantly higher than the third day and the first day (before the intervention) (P < 0.001). The mean weight of preterm infants on the third day in the intervention, placebo and control groups was significantly higher than the first day (before the intervention) (P < 0.001).

## Discussion

Within this first study of its kind we aimed to determine the effect of oral consumption of *Lactuca sativa* (*L. sativa*) syrup (lettuce extract) on human milk volume and subsequent weight gain in the preterm infant. In terms of the variables compared between the groups in the present study, the variables of mode of birth, birth weight and height of the infant, IUGR and the type of serum injected into the infants were significant between the three groups. For this reason, the analysis was performed once with the control of the effect of intervening variables and once without.

Excluding the first day, the mean volume of human milk pumped showed a statistically significant difference between the groups. The mean volume of pumped milk in the test group was higher than the other two groups. Using covariance analysis, adjusting the variables related to mode of birth, infant birth weight, IUGR and the volume of milk pumped the previous day, a statistically significant difference was observed on the fourth and fifth day of the intervention. Although the intervention group had a higher adjusted mean volume of pumped milk, this difference was not statistically significant between the three groups on the seventh day.

As this is the first study of its kind, articles presenting comparative results remain unavailable. Nevertheless, in Ibn Sina's book (Canon medicine) and according to evidence-based practical reports of Nice in 2015, lettuce is one of the things that is known to increase milk production^33,35,36^. Lettuce contains phytoestrogens^34^, flavonoid compounds^37^ and Lignans. Lignan also has estrogenic properties^31^, and the ethanol extract of lettuce causes and increases the synthesis of sex hormones including estradiol and prolactin^30^. Considering that prolactin is the most important hormone related to human milk feeding^49^, and stimulating the production and expression of its receptor is the responsibility of estrogen^50,51^, it can be assumed that lettuce has milk-enhancing properties due to the presence of phytoestrogens. Thus, the results presented here should not be considered surprising.

Lettuce also contains flavonoid compounds^37^, and these flavonoids have analgesic and anti-inflammatory properties which work through various mechanisms^52^ Moreover, lettuce interacts with opioid receptors^38,39^ and opioids, by acting on mast cells, causing degranulation, histamine release, and finally vasodilation^40^. As such, lettuce may also increase blood supply to mammary glands through vasodilation and ultimately increase milk production. The nature of lettuce is also cold and moist, and it has diuretic, laxative, moisturizing and thirst-quenching effects^40–42^. Consequently, lettuce may similarly increase the secretion of body fluids such as milk. There are also further studies confirming the estrogenic (phytoestrogen) effects of lettuce. For example, due to the presence of Lignan (one of the main components of the family of phytoestrogens) and its precursors, lettuce has a further protective effect against breast cancer in premenopausal cisgender women^32^ and against esophageal cancer in humans^31^ .These mechanisms may further provide context to the results presented here.

Other plants such as fenugreek, silymarin (milk thistle) and ginger have lactating properties. Fenugreek has milk-enhancing properties due to its phytoestrogenic structure (flavonoids, terpenoids and saponin)^48^ and its effectiveness has been shown in several studies^47,48,53^. Silymarin is rich in flavonoid compounds that stimulate the secretion of prolactin, and flavonolignans are flavonoid phytoestrogens that directly stimulate lactation^54–56^. Ginger also causes vascular dilation^57^ and warms the ambient temperature. The warming of the ambient temperature is likely due to the dilation of blood vessels. This mechanism in particular may explain the increase in milk production through increased blood supply to the mammary glands^58^. Yet unlike in these alternative plant groups, our participants reported no adverse side effects.

Results presented here demonstrate no statistically significant difference in terms of the mean changes (with or without adjustment) in the weight gain of premature infants in all three groups. Thus there remains no evidence to support the effectiveness of *Lactuca sativa* (*L. sativa*) syrup (lettuce extract) on the weight gain of premature infants. Fenugreek has same effective mechanism as lettuce, and has similarly been tested and found to have no statistically significant difference between the groups in terms of infant weight gain^28,47^. The infants’ age at birth and/or the short-term intervention period may somewhat explain why the effectiveness of Sativa (*L. sativa*) syrup in promoting weight gain in the preterm infant was not seen in the present study. Weight gain in premature infants is typically slower than that of full-term infants, and they also have different nutritional needs. Premature infants may also have poorer weight gain where parents have lower levels of literacy^59^. As such, it is worth noting that approximately only 10% of the adult participants included in the present study had a university education. Whilst we have not been able to provide evidence of the effectiveness of lettuce on premature infant weight gain via human milk, neither have other studies exploring a variety of other herbs^27^ . Thus, the pursuit of effective interventions remains.

For context, *Lactuca sativa* syrup (Noma) is made in Iran and approved by the Food and Drug Organization with the registration number 0545–95-س, is produced and made available and affordable by the pharmaceutical company "Sanabel Daru". It is marketed as a sedative and sleep inducer. It also helps to treat hyperactivity in children^60^. In this first study of its kind, there were no side effects caused by the oral consumption of *Lactuca sativa* syrup in those lactating, and human milk feeding their preterm infants. Therefore, its use for infant feeding and increased milk production is acceptable in terms of safety.

### Strengths and limitations

The main strength of this study is that it is the first to examine the effect of *Lactuca sativa* syrup on the volume of human milk and subsequent weight gain of preterm infants. Future studies are encouraged to use other medicinal forms of *Lactuca sativa* (e.g., tea, capsule, powder) with varying doses. Due to the spread of COVID-19, use of electric human milk pumps may have caused anxiety and confusion in participants. Yet face-to-face hygiene training and individual training via WhatsApp along with the ability to contact the research team with concerns controlled this to some extent. We recognize that there are individual differences in human milk production. Yet by randomly assigning the participants to the study groups, we may also have reduced this effect to some extent. This study was restricted to a 7-day intervention period, as preterm infants are usually fed human milk directly after this time (instead of tube feeding), in which case accurate recording of the volume of milk produced is no longer possible. Consequently, it is suggested that future research explore the effectiveness of *Lactuca sativa* syrup on the weight gain of infants born at < 37 weeks gestation.

## Conclusion

The results of this foundational study demonstrate that the mean volume of human milk produced after adjusting the significant variables at the end of the study (after 1 week) was significantly higher in the intervention group than the control group. Conversely, there was no statistically significant difference observed in the mean weight gain of preterm infants (after 1 week) between intervention and control groups. Moreover, there was absence of side effects caused by the consumption of *Lactuca sativa* syrup in this context. Therefore, *Lactuca sativa* syrup may be used orally as an affordable, easily accessible, safe and effective method to increase the volume of milk in humans.

## Data Availability

All the information obtained from this study is not available to the public due to the confidentiality of the information, but it can be made available upon reasonable request through the Corresponding author.
